# Dose-Dependent Effects of a Soluble Dietary Fibre (Pectin) on Food Intake, Adiposity, Gut Hypertrophy and Gut Satiety Hormone Secretion in Rats

**DOI:** 10.1371/journal.pone.0115438

**Published:** 2015-01-20

**Authors:** Clare L. Adam, Patricia A. Williams, Karen E. Garden, Lynn M. Thomson, Alexander W. Ross

**Affiliations:** Ingestive Behaviour Group, Obesity & Metabolic Health Division, Rowett Institute of Nutrition & Health, University of Aberdeen, Aberdeen AB21 9SB, Scotland, United Kingdom; National Institute of Agronomic Research, FRANCE

## Abstract

Soluble fermentable dietary fibre elicits gut adaptations, increases satiety and potentially offers a natural sustainable means of body weight regulation. Here we aimed to quantify physiological responses to graded intakes of a specific dietary fibre (pectin) in an animal model. Four isocaloric semi-purified diets containing 0, 3.3%, 6.7% or 10% w/w apple pectin were offered *ad libitum* for 8 or 28 days to young adult male rats (*n* = 8/group). Measurements were made of voluntary food intake, body weight, initial and final body composition by magnetic resonance imaging, final gut regional weights and histology, and final plasma satiety hormone concentrations. In both 8- and 28-day cohorts, dietary pectin inclusion rate was negatively correlated with food intake, body weight gain and the change in body fat mass, with no effect on lean mass gain. In both cohorts, pectin had no effect on stomach weight but pectin inclusion rate was positively correlated with weights and lengths of small intestine and caecum, jejunum villus height and crypt depth, ileum crypt depth, and plasma total glucagon-like peptide-1 (GLP-1) and peptide tyrosine tyrosine (PYY) concentrations, and at 8 days was correlated with weight and length of colon and with caecal mucosal depth. Therefore, the gut’s morphological and endocrine adaptations were dose-dependent, occurred within 8 days and were largely sustained for 28 days during continued dietary intervention. Increasing amounts of the soluble fermentable fibre pectin in the diet proportionately decreased food intake, body weight gain and body fat content, associated with proportionately increased satiety hormones GLP-1 and PYY and intestinal hypertrophy, supporting a role for soluble dietary fibre-induced satiety in healthy body weight regulation.

## Introduction

In the battle to prevent overweight and obesity, satiety-inducing food ingredients such as dietary fibre offer a natural dietary strategy for caloric intake control and body weight regulation [1]. We have recently used an animal model to demonstrate significant reductions in voluntary food intake, body weight gain and adiposity when young adult rats were fed diets containing a range of different soluble fermentable dietary fibres, including pectin [2]. The results indicated that the presence of soluble fibre was more critical than its source, and this follow-on study addressed the potential importance of the amount of soluble fibre consumed. Pectin was the highly fermentable soluble dietary fibre used herein; it is a plant cell structural heteropolysaccharide, naturally occurring in fruit and vegetables, but also commercially extracted, available as a food supplement and widely used by the food industry [3]. In our earlier rat study, 10% w/w dietary pectin for 4 weeks increased satiety, decreased food intake, decreased body weight gain and led to loss of body fat [2]. Since this dietary inclusion rate equated to approximately twice the dietary fibre RDA for American men [4, 5], lower inclusion rates were included in the present study.

Increased soluble dietary fibre consumption increases circulating concentrations of the gut satiety hormones glucagon-like peptide-1 (GLP-1) and peptide tyrosine tyrosine (PYY) in rats [2, 6–8]. There is some evidence to indicate that appetite and food intake suppression by these peripheral hormones is dose-sensitive [9–11] but it is less clear whether the stimulation of these hormones by soluble dietary fibre is also dose-sensitive. In support, gene expression for PYY and GLP-1 was dose-dependently increased in rats given dietary prebiotics [12]. However, whilst dietary oligofructose dose-dependently increased circulating PYY in a human study, it did not significantly affect GLP-1, appetite ratings or caloric intake [13]. After 4 weeks of increased soluble dietary fibre in our rat model, the increased circulating concentrations of total GLP-1 and PYY both correlated with the decreased food intake [2]. Here we sought to determine the dose dependent effects of the soluble fibre pectin on both circulating satiety hormone concentrations and food intake.

It has long been noted that rats fed increased amounts of dietary fibre, including pectin, show increased gut weights and increased intestinal mucosal cell proliferation [14–16] and more recently the ingestion of prebiotic fibres has been associated with increased gut weight and length [12]. Increased gut size was observed (but not measured) previously in our model [2]. Since changes to gut morphometry may ultimately influence the efficacy, safety and acceptance of high soluble fibre diets for human body weight control, it is important to examine this phenomenon more closely. Here we aimed for the first time to quantify this response by weighing and measuring gut regions after different doses of increased dietary fibre and to characterise the response by taking histological measurements of regional mucosal depth.

It is axiomatic that the mammalian gut needs time to adapt to changes in diet composition. After the abrupt introduction of soluble fibre to the daily diet in our rat model, there followed a period of adaptation lasting approximately a week before voluntary food intake stabilised at a steady level for the next 3 weeks [2]. Here we sought to examine whether changes in gut morphometry and/or endocrinology occurred during this adaptation period and so we examined responses in the short term (~1 week) in addition to the longer term (4 weeks).

Therefore, the purpose of the present study was to examine the dose dependent effects of a soluble fermentable dietary fibre (pectin) on food intake, body weight and body composition in young adult male rats. Additional outcome measures following either 8 or 28 days of pectin consumption included plasma concentrations of satiety hormones, PYY and GLP-1, and characterisation of the changes in gut morphology and histology.

## Materials and Methods

### Ethics statement

All animal experimental procedures met institutional and national guidelines for the care and use of animals. They were licensed by the UK Home Office Animals (Scientific Procedures) Act, Amended 2012, under Project License 60/4282 and were approved by the local ethical review committee at the University of Aberdeen Rowett Institute of Nutrition & Health (approval number SA12/04E). The rats were euthanised by decapitation under general inhalation anaesthesia (isoflurane; IsoFlo, Abbott Animal Health, Maidenhead, Berkshire, UK).

### Diets

All diets were based on the purified AIN-93M diet (American Society for Nutrition, Bethesda, MD, USA) for the maintenance of adult rats and were made and supplied by Special Diet Services Ltd, Witham, Essex, UK. The isocaloric experimental diets (15.7 MJ/kg, calculated from the standard energy values for carbohydrate, protein, fat and fibre of 16.7, 16.7, 37.6 and 8.4 MJ/kg respectively) contained 3.3%, 6.7% or 10% w/w soluble pectin fibre (apple pectin; Solgar Apple Pectin, Revital Ltd., Ruislip, Middlesex UK) (3%P, 7%P and 10%P groups, respectively) while the control diet (C) contained 0% pectin (Table 1). The insoluble dietary fibre cellulose was included in the C diet and for balancing total fibre in the experimental diets as appropriate.

**Table 1:** Composition of experimental diets (%w/w).

	**Diet**			
	**CONT**	**3%P**	**7%P**	**10%P**
Maize starch	46.57	41.57	41.57	41.57
Maltodextrin	15.5	15.5	15.5	15.5
Sucrose	10	10	10	10
Casein	14	14	14	14
Soyabean oil	4	4	4	4
AIN-93 Mineral mix	3.5	3.5	3.5	3.5
AIN-93 Vitamin mix	1	1	1	1
Choline bitartrate	0.25	0.25	0.25	0.25
L-cystine	0.18	0.18	0.18	0.18
Cellulose	5	6.7	3.3	0
Pectin^1^	0	3.3	6.7	10.0

All diets are based on AIN-93M diet (American Society for Nutrition, Bethesda, MD USA).

^1^ Apple pectin (Solgar Apple Pectin; Revital Ltd., Ruislip, Middlesex UK).

### Animals, experimental procedure and tissue collection

After 1 week acclimatisation to individual housing in plastic cages while on C diet, 64 outbred male Sprague Dawley rats (12 weeks old, 444.5 ± 3.70 g; from Charles River Laboratories UK) were offered the pelleted experimental diets *ad libitum* for either 8 days or 28 days (*n* = 8/group). Water was available *ad libitum*, the lighting regime was a standard 12 h light and 12 h dark, temperature was constant at 21±2°C and the relative humidity was held at 55±10%; cages contained sawdust bedding with shredded paper for nesting and plastic tunnels for further environmental enrichment. Voluntary food intake was measured daily by weighing uneaten food each morning and body weight was measured twice a week. Body composition was measured in conscious rats at the start (day 0) and end (8 days or 28 days) of the experiment by magnetic resonance imaging (MRI; EchoMRI 2004, Echo Medical Systems, Houston, TX, USA), which provided total body fat and lean mass data.

After the final MRI scan, rats in both 8-day and 28-day cohorts were euthanised approximately 1–3 h after lights on. Final (trunk) blood samples were collected into chilled tubes containing EDTA as anti-coagulant and a peptidase inhibitor cocktail containing general protease inhibitor (cØmplete; Roche Diagnostics Ltd, Burgess Hill, West Sussex, UK) and dipeptidyl peptidase-4 inhibitor (Ile-Pro-Ile; Sigma-Aldrich, Gillingham, Dorset, UK), centrifuged immediately at 3000g for 10 min, then plasma stored at -20°C until analysis. The gut was dissected out, wet weights were recorded immediately for whole gut, stomach, small intestine, caecum and colon, and the lengths of small intestine, caecum and colon were measured. Tissue samples from jejunum, distal ileum, caecum and proximal and distal colon were frozen on dry ice in Tissue-Tek (O.C.T. Compound, Sakura Finetek UK Ltd., Thatcham, Berkshire, UK) and stored at -80°C.

### Gut histology

Cross sectional cryostat sections (20 µm) of jejunum, distal ileum, caecum, proximal and distal colon were mounted on microscope slides and stained with haemotoxylin and eosin using standard methodology (reagents from Sigma-Aldrich). Measurements were taken of villus height and crypt depth in the jejunum and ileum, and overall depth of mucosa in the caecum, proximal and distal colon, using Image Pro-plus software (Media Cybernetics Inc., Rockville, MD, USA) linked to a light microscope (Leica DMR; Leica Microsystems (UK) Ltd., Milton Keynes, UK). Up to 10 measurements were taken at 5x magnification in vertical cross sectional plane at different sites from 2 sections per gut region per rat in order to provide an average value for each parameter for each individual.

### Plasma analyses

Plasma samples were analysed by commercial RIA kits according to the manufacturers’ instructions. Total GLP-1 was measured by kit GLP1T-36HK (Merck Millipore, Billerica, MA, USA; lower detection limit 3 pM) which detects all forms of GLP-1. Active GLP-1 was not measured because it has a short half-life in plasma, and measurement of total GLP-1 provides an accurate indication of overall GLP-1 secretion since it includes both the intact hormone and its primary metabolite [17]. PYY was measured by kit RMPYY-68HK (Merck Millipore; lower detection limit 15.6 pg/ml), which detects both of the circulating biologically active forms of PYY, namely PYY(1–36) and PYY(3–36).

### Statistical methods

Daily food intakes and twice weekly body weight data were analysed within 8-day and 28-day cohorts by repeated measures ANOVA (General Linear Model (GLM) with time, diet and their interaction as factors; Minitab Version 16, Minitab Inc., State College, PA). Group comparisons for all other data were performed by ANOVA (one-way or GLM with cohort, diet and their interaction as factors as appropriate) followed by Tukey’s post-hoc tests, and Pearson’s product-moment correlation was used to examine relationships between measured parameters where indicated (Minitab). *P* values of 0.05 or less were considered statistically significant.

## Results

### Food intake, body weight and body composition

**Figure 1:**
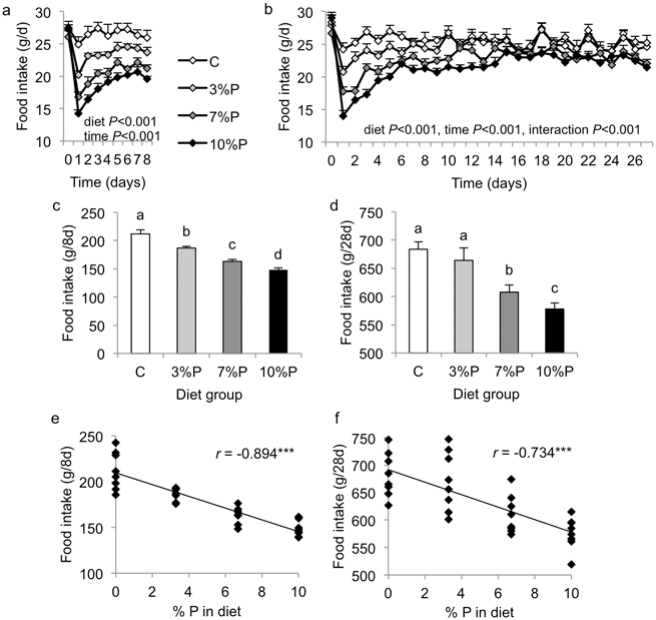
Food intake. Daily (a, b) and cumulative (c, d) voluntary food intake by rats offered control diet (C) or diet containing 3, 7 or 10% pectin (P) for 8 days (a, c) or 28 days (b, d), and correlation between cumulative intake and amount of P in the diet in 8-day (e) and 28-day (f) cohorts. Results of repeated measures ANOVA are indicated in figures a and b. Columns labelled with different letters in figures c and d are significantly different by one-way ANOVA, *P*<0.05, and *r* is the Pearson product-moment correlation coefficient in figs e and f, ***P<0.001.

Daily food intake diverged immediately between the diet groups (Fig. 1a,b). Repeated measures ANOVA revealed significant effects of diet (*P*<0.001) and time (*P*<0.001) for both cohorts, with a significant diet x time interaction (*P*<0.001) only for the 28-day cohort. Daily intakes were significantly different between all four groups in the 8-day cohort, C > 3%P > 7%P > 10%P, and were C = 3%P > 7%P > 10%P in the 28-day cohort (Fig. 1a,b). Importantly, repeated measures ANOVA from day 14 onwards for the 28-day cohort (i.e. excluding the early period of very low intakes) revealed similarly significant effects of diet on daily food intake (C = 3%P>7%P>10%P; *P*<0.001). Compared with respective C groups, cumulative intake by the 8-day cohort was decreased in 3%P (*P*<0.01), 7%P (*P*<0.001) and 10%P (*P*<0.001) groups and by the 28-day cohort was decreased in 7%P (*P*<0.01) and 10%P (*P*<0.001) groups (Fig. 1c,d). Overall, cumulative intake in both cohorts showed highly significant negative correlation with the amount of pectin in the diet (both *P*<0.001; Fig. 1e,f).

**Figure 2:**
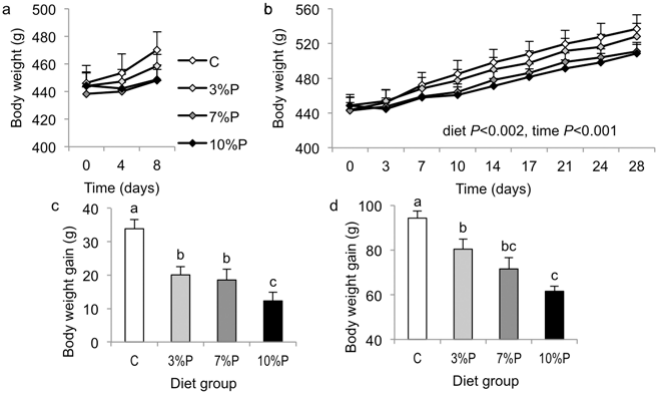
Body weight. Changes in body weight in rats offered control diet (C) or diet containing 3, 7 or 10% pectin (P) for 8 days (a, c) or 28 days (b, d). Results of repeated measures ANOVA are indicated in fig b. Within figures c and d, columns labelled with different letters are significantly different by one-way ANOVA, *P*<0.05.

**Figure 3:**
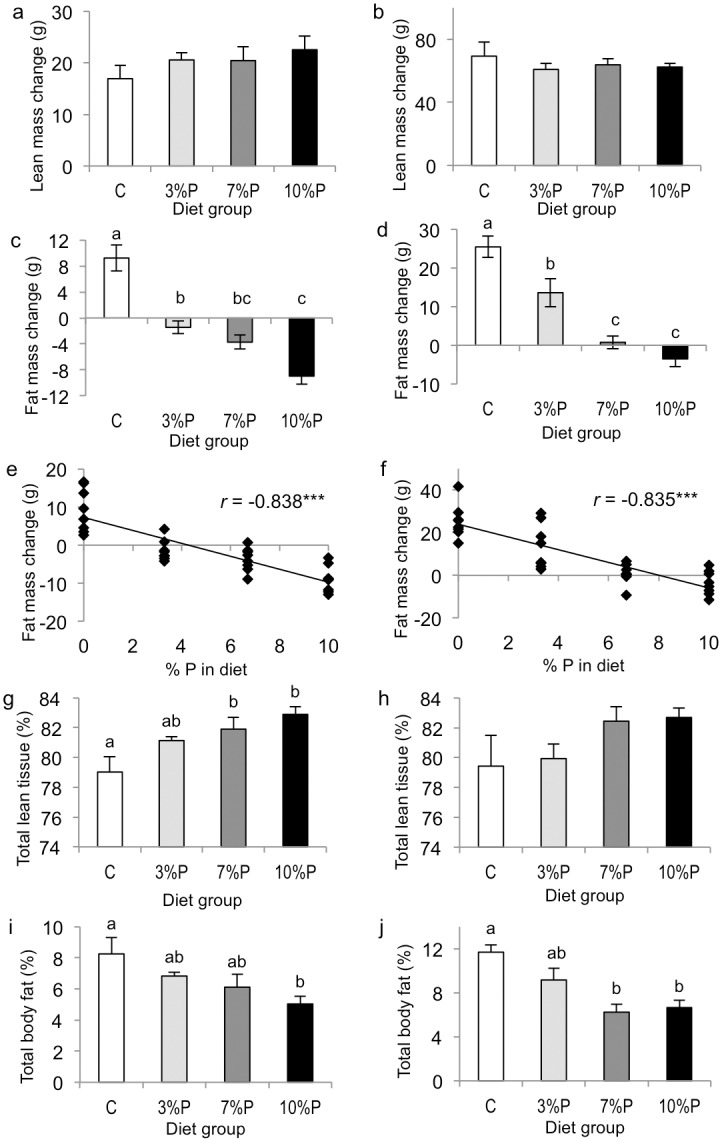
Body composition (by MRI). Changes in total lean tissue mass (a, b) and total fat mass (c, d), correlation between change in body fat mass and amount of pectin in the diet (e, f), final total body lean tissue percentage (g, h), and final total body fat percentage (i, j) in rats offered control diet (C) or diet containing 3, 7 or 10% pectin (P) for 8 days (a, c, e, g, i) or 28 days (b, d, f, h, j). Within figures, columns labelled with different letters are significantly different by one-way ANOVA, *P*<0.05, and *r* is the Pearson product-moment correlation coefficient, ***P<0.001.

Repeated measures ANOVA revealed significant effects of diet (*P*<0.002) and time (*P*<0.001) on twice-weekly body weights for the 28-day cohort but no effects for the 8-day cohort (Fig. 2a,b). However, compared with their respective C groups, overall body weight gain was decreased in 3%P (*P*<0.01), 7%P (*P*<0.01) and 10%P (*P*<0.001) groups in the 8-day cohort and in 3%P (*P*<0.01), 7%P (*P*<0.01) and 10%P (*P*<0.001) groups in the 28-day cohort (Fig. 2c,d); and body weight gain correlated negatively with the amount of pectin in the diet in both cohorts (*r* = -0.688 and -0.752, respectively, both *P*<0.001). Total lean tissue gain was not different between the groups in either cohort (Fig. 3a,b). In the 8-day cohort, total body fat mass increased in group C, showed no change in 3%P (*P*<0.01 compared with C) but decreased in 7%P and 10%P groups (both *P*<0.001 compared with C; Fig. 3c). In the 28-day cohort, total fat mass increased in group C, showed a smaller increase in 3%P (*P*<0.01 compared with C), no change in 7%P (*P*<0.001) and a decrease in the 10%P group (*P*<0.001; Fig. 3d). In both cohorts the increase in total body fat mass correlated negatively with the amount of pectin in the diet (both *P*<0.001; Fig. 3e,f). Final total body lean tissue percentage was higher in 7%P and 10%P groups than group C in the 8-day cohort (*P*<0.01) but was not different between groups in the 28-day cohort (Fig. 3g,h). Final total body fat percentage was lower in 10%P (*P*<0.01) than group C in the 8-day cohort and lower in 7%P and 10%P (both *P*<0.001) than group C in the 28-day cohort (Fig. 3i,j).

### Gut morphology

Overall weights of whole gut (9%, *P*<0.01) and caecum (24%, *P*<0.001) and length of small intestine (4%, *P*<0.01) were greater in the 28-day rats than in the 8-day cohort, but weights of stomach, small intestine and colon and lengths of caecum and colon were not different; there were no cohort x diet interactions and the data are presented separately for each cohort in Table 2. Compared with their respective C groups, whole gut weight was greater in 7%P (*P*<0.01) and 10%P (*P*<0.001) groups in the 8-day cohort and in the 10%P group in the 28-day cohort (*P*<0.01); within each cohort whole gut weight correlated significantly with the amount of pectin in the diet (both *P*<0.001; Table 2). Stomach weight was not different between the groups in either cohort. Compared with their respective C groups, small intestine weight was greater in 3%P (*P*<0.01), 7%P and 10%P groups (both *P*<0.001) in the 8-day cohort and the 7%P and 10%P groups in the 28-day cohort (both *P*<0.001); within each cohort small intestine weight correlated significantly with the amount of pectin in the diet (both *P*<0.001; Table 2). Similarly, in both 8-day and 28-day cohorts, small intestine length was increased in 7%P (*P*<0.05 and *P*<0.01, respectively) and 10%P groups (*P*<0.01 and *P*<0.001, respectively) and correlated significantly with the amount of pectin in the diet (both *P*<0.001; Table 2). Caecum weight was greater in 3%P (*P*<0.01), 7%P (*P*<0.001) and 10%P groups (*P*<0.001) than the C group within each cohort and correlated significantly with the amount of pectin in the diet (both *P*<0.001; Table 2). Similarly caecum length was greater in 3%P (*P*<0.01), 7%P (*P*<0.001) and 10%P groups (*P*<0.001) within each cohort and correlated significantly with the amount of pectin in the diet (both *P*<0.001; Table 2). In the 8-day cohort, colon weight was greater in 7%P and 10%P groups than the C group (both *P*<0.01) and correlated significantly with the amount of pectin in the diet (*P*<0.001); in the 28-day cohort, colon weight was greater in 3%P and 7%P groups than the C group (both *P*<0.01) and did not correlate with the amount of pectin in the diet (Table 2). In the 8-day cohort, colon length was greater in the 10%P group than C (*P*<0.01) and correlated with the amount of pectin in the diet (*P*<0.001), while in the 28-day cohort there were no group differences in colon length and no correlation (Table 2).

**Table 2:** Gut regional weights, lengths and histological measurements in rats fed control diet (C) or diet containing 3, 7 or 10% pectin (P) for 8 days or 28 days.

	**8-day cohort diet groups**					**28-day cohort diet groups**				
	**C**	**3%P**	**7%P**	**10%P**	***r***	**C**	**3%P**	**7%P**	**10%P**	***r***
*Gut morphology*										
Whole gut weight (g)	27.7^a^ ±1.5	30.6^ab^ ±1.3	34.4^bc^ ±1.1	36.6^c^ ±1.0	0.716***	30.6^a^ ±1.5	35.0^ab^ ±1.4	35.8^ab^ ±1.2	39.2^b^ ±1.9	0.589***
Stomach weight (g)	6.3 ±0.5	5.4 ±0.4	6.5 ±0.7	5.2 ±0.3	-0.153	4.8 ±0.8	6.0 ±0.8	6.1 ±0.7	5.8 ±0.7	0.168
Small intestine weight (g)	8.4^a^ ±0.4	10.0^b^ ±0.4	11.6^c^ ±0.3	13.8^d^ ±0.3	0.910***	9.3^a^ ±0.4	10.5^ab^ ±0.3	11.6^b^ ±0.4	13.3^c^ ±0.5	0.808***
Small intestine length (mm)	1043^a^ ±14	1057^ab^ ±13	1123^bc^ ±21	1149^c^ ±29	0.617***	1086^a^ ±11	1105^ab^ ±13	1154^bc^ ±19	1184^c^ ±16	0.688***
Caecum weight (g)	3.0^a^ ±0.2	4.7^b^ ±0.2	5.4^b^ ±0.4	7.0^c^ ±0.5	0.834***	3.5^a^ ±0.2	5.4^b^ ±0.3	6.8^b^ ±0.5	9.3^c^ ±0.6	0.876***
Caecum length (mm)	37.5^a^ ±0.9	44.1^b^ ±1.9	50.6^c^ ±1.7	56.9^d^ ±1.3	0.874***	35.9^a^ ±1.1	44.4^b^ ±1.5	52.8^c^ ±1.9	59.4^d^ ±1.7	0.902***
Colon weight (g)	2.4^a^ ±0.2	3.6^ab^ ±0.3	4.1^b^ ±0.5	4.4^b^ ±0.4	0.560***	2.3^a^ ±0.3	4.2^b^ ±0.3	3.9^b^ ±0.5	3.5^ab^ ±0.4	0.297
Colon length (mm)	157^a^ ±4	176^ab^ ±7	183^ab^ ±9	194^b^ ±8	0.561***	166 ±5	171 ±2	174 ±4	174 ±6	0.157
*Gut histology* (µm)										
Jejunum villus height	583.5 ±47.2	612.3 ±15.2	647.0 ±21.1	685.9 ±18.9	0.499*	539.9 ±25.5	630.7 ±40.1	661.2 ±39.9	672.1 ±63.7	0.483*
Jejunum crypt depth	172.1 ±17.0	159.7 ±6.1	195.4 ±17.5	216.2 ±16.4	0.440*	149.2^a^ ±9.5	158.5^a^ ±8.6	183.8^a^ ±16.2	292.5^b^ ±16.8	0.815***
Distal ileum villus height	263.5 ±20.7	335.8 ±34.3	382.4 ±48.8	311.6 ±25.0	0.173	295.9 ±11.8	315.9 ±23.3	281.3 ±6.5	338.7 ±20.6	0.205
Distal ileum crypt depth	150.0^a^ ±8.2	158.5^a^ ±11.2	213.8^b^ ±13.4	179.8^ab^ ±13.6	0.491*	150.7^a^ ±12.7	153.3^a^ ±16.1	172.7^a^ ±12.4	263.4^b^ ±27.4	0.662***
Caecum mucosa depth	184.3^a^ ±8.3	188.6^a^ ±7.7	209.6^ab^ ±7.6	231.8^b^ ±7.2	0.681***	209.5 ±15.1	206.4 ±7.9	217.8 ±12.4	215.8 ±8.2	0.115
Proximal colon mucosa depth	151.7 ±8.4	193.6 ±17.1	176.2 ±9.7	188.8 ±13.7	0.241	178.7 ±12.7	209.8 ±19.5	210.1 ±10.0	210.2 ±9.3	0.308
Distal colon mucosa depth	215.7 ±6.0	221.8 ±21.5	236.2 ±21.2	230.4 ±14.9	0.195	224.8 ±6.9	238.0 ±11.8	252.2 ±16.1	251.1 ±11.0	0.340

Values are means ± s.e.m. (*n* = 8/group). Within rows and cohorts, means with different superscript letters are significantly different, P<0.05. *r*, correlation with %P (Pearson correlation coefficient; *n* = 32/cohort);

*** *P*<0.001,

* *P*<0.05.

### Gut histology

Overall, gut histology measurements were not significantly different between the cohorts except for mucosal depth in the proximal colon which was greater in 28-day than 8-day rats (14%, *P*<0.01). However, there were overall significant effects of pectin-containing diets increasing mucosal depth in the caecum (*P*<0.05) and proximal colon (*P*<0.05), increasing crypt depth in the jejunum (*P*<0.001) and distal ileum (*P*<0.001) and villus height in the jejunum (*P*<0.01). There were significant cohort x diet interactions for crypt depth in the jejunum and distal ileum (both *P*<0.001) since the dietary effect was greater in the 28-day cohort. Data are presented separately for each cohort in Table 2. Compared with their respective C groups, in the 8-day cohort ileum crypt depth was significantly greater in the 7%P group (*P*<0.001) and caecum mucosa thickness was greater in the 10%P group (*P*<0.001), while in the 28-day cohort jejunum and ileum crypt depths were greater in the 10%P group (both *P*<0.001, Table 2). Correlation analysis revealed significant positive relationships with the amount of dietary pectin in both the 8-day and 28-day cohorts for jejunum villus height (both *P*<0.05), jejunum crypt depth (*P*<0.05 and *P*<0.001, respectively) and distal ileum crypt depth (*P*<0.05 and *P*<0.001, respectively) and in the 8-day cohort only for caecum mucosal depth (*P*<0.001; Table 2).

### Plasma satiety hormone concentrations

**Figure 4:**
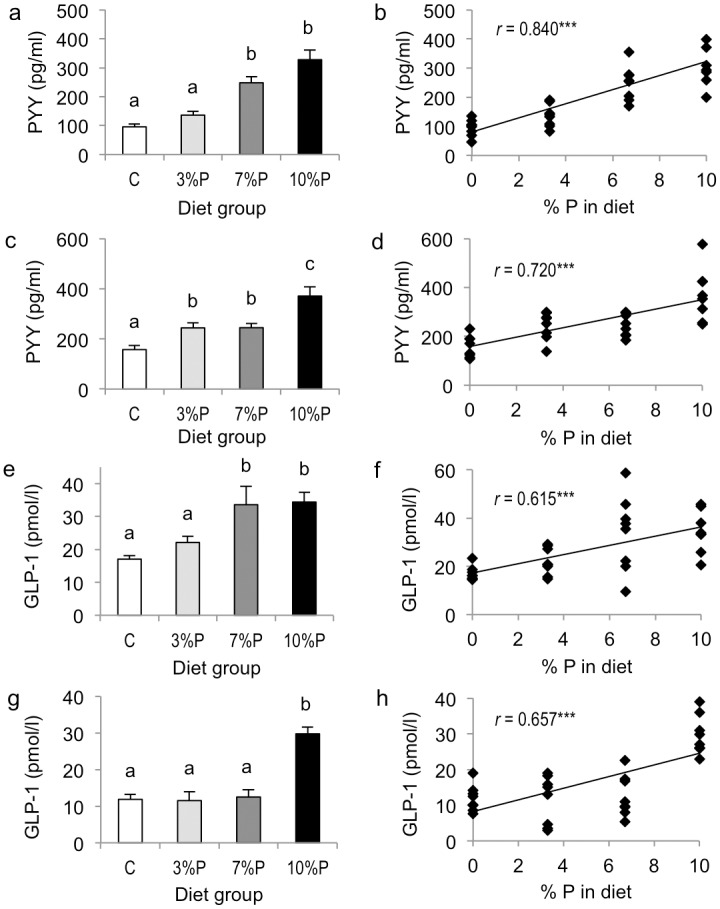
Satiety hormones. Plasma concentrations of PYY (a, c) and total GLP-1 (e, g) in rats offered control diet (C) or diet containing 3, 7 or 10% pectin (P) for 8 days (a, e) or 28 days (c, g); and correlations between amount of pectin in the diet and PYY and total GLP-1 concentrations in the 8-day (b, f) and 28-day (d, h) cohorts. Within figures, columns labelled with different letters are significantly different by one-way ANOVA, *P*<0.05, and *r* is the Pearson product-moment correlation coefficient, ***P<0.001.

There was no difference between the cohorts for plasma PYY concentrations, but significant effects of diet. Compared with respective C groups, plasma PYY concentrations were increased in 7%P and 10%P groups in the 8-day cohort (both *P*<0.001; Fig. 4a) and in 3%P, 7%P and 10%P groups in the 28-day cohort (*P*<0.05–0.001; Fig. 4c). In the 8-day cohort, plasma PYY correlated with the amount of pectin in the diet (*P*<0.001; Fig. 4b) and with the weights of small intestine (*r* = 0.794, *P*<0.001), caecum (*r* = 0.721, *P*<0.001) and colon (*r* = 0.444, *P*<0.01), and correlated negatively with cumulative food intake (*r* = -0.745, *P*<0.001). Similarly, in the 28-day cohort, plasma PYY correlated with the amount of pectin in the diet (*P*<0.001; Fig. 4d) and with the weights of small intestine (*r* = 0.720, *P*<0.001) and caecum (*r* = 0.723, *P*<0.001), but not colon (*r* = 0.240), and correlated negatively with cumulative food intake (*r* = -0.451, *P*<0.01).

There were effects of cohort (8-day>28-day, *P*<0.01) and diet (*P*<0.001) and cohort x diet interaction (*P*<0.001) for plasma total GLP-1 concentrations. Compared with respective C groups, plasma total GLP-1 concentrations were increased in 7%P and 10%P groups in the 8-day cohort (both *P*<0.001; Fig. 4e) and in the 10%P group in the 28-day cohort (*P*<0.001; Fig. 4g). In both the 8-day and 28-day cohorts, plasma total GLP-1 correlated with the amount of pectin in the diet (both *P*<0.001; Fig. 4f,h) and with the weights of small intestine (*r* = 0.583 and 0.575, respectively; both *P*<0.001) and caecum (*r* = 0.596 and 0.674, respectively; *P*<0.001), but not colon (*r* = 0.193 and 0.000, respectively). Plasma total GLP-1 correlated negatively with cumulative food intake in both the 8-day and 28-day cohorts (*r* = -0.585 and -0.541, respectively; *P*<0.001) and positively with plasma PYY concentrations (*r* = 0.816 and 0.681, respectively; both *P*<0.001).

## Discussion

This study has demonstrated in an animal model that increasing amounts of the soluble fibre pectin in the diet proportionately decreased food intake, body weight gain and body fat content, and proportionately increased gut size and circulating PYY and total GLP-1 concentrations, in both the short- and long-term. The data are consistent with PYY and GLP-1 being involved in mediating dietary fibre-induced satiety and demonstrate how the gut’s morphological and endocrine adaptations are dose-sensitive, occur within 8 days and are sustained over at least 28 days during continued dietary intervention.

The present results agree with the dose-dependently decreased caloric intake reported in rats given up to 20% dietary inulin:oligofructose for 10 weeks [12] although increasing doses of a mixed cocktail of soluble dietary fibres were found to have no effect on food intake 3 h later in humans [18], the difference most likely being attributable to the very short-term design of the latter study. Importantly, the reductions in food intake herein were accompanied by significant decreases in body weight gain and specifically prevented fat mass gain whilst maintaining normal lean tissue gain.

At 12 weeks of age the present rats were young adults, having ceased the juvenile rapid growth phase but nonetheless continuing to gain body mass at a slower rate [19]. This rate of gain was decreased throughout the 28-day experiment in inverse proportion to the amount of soluble fibre in the diet. Although the immediate response was likely due to the abrupt decline in food intake upon introduction of pectin to the experimental diets, the effect was nonetheless sustained. Importantly, after both 8 and 28 days, there was no effect of soluble dietary fibre on lean tissue gain and the differences in body weight gain were entirely attributable to a dose-dependent inhibition of body fat gain. This indicates that soluble dietary fibre may be particularly suitable for healthy body weight regulation in humans. Here the greatest reductions in body weight and body fat gain occurred at the highest dose of 10% pectin w/w, or approximately 6.6mg/kJ, but were nonetheless significant (by extrapolation) at 5% w/w, or 3.3 mg/kJ, which is the recommended daily fibre intake for US men [4, 5]. Thus practicable levels of soluble dietary fibre have clear potential to limit weight gain by limiting body fat accumulation whilst maintaining lean mass, and this approach is worthy of further investigation in humans. In one human study using dietary pectin (10g) for 15 days satiety ratings were increased in women but effects on caloric intake were inconsistent and there was no effect on body weight [20]; however this dose rate was low (equivalent to about 1.2 mg/kJ) compared with the present findings and higher doses for humans may reveal more positive results.

The decrease in food intake was interpreted here as a measure of increased satiety since intakes correlated with circulating concentrations of satiety hormones PYY and GLP-1, as observed previously in soluble fibre-fed young adult rats [2] but now also seen across different fibre doses and time points. In support, peripheral PYY administration dose-dependently inhibits food intake in rats [9, 10] and food intake inhibition by peripheral GLP-1 is linearly related to the dose infused in humans [21]. Pectin consumption dose-dependently increased tonic secretion of both of these hormones in the present study after both 8 and 28 days, with PYY showing the closer relationship (Fig. 4). Similarly, dietary inulin:oligofructose for 10 weeks dose-dependently increased the active GLP-1 response to a meal and intestinal mRNA levels of both proglucagon (GLP-1 precursor) and PYY in rats [12]. These hormones are co-secreted (reflected here in the close correlation between their plasma concentrations) by enteroendocrine L-cells, which are distributed fairly evenly along the rat jejunum, ileum and colon [22]. The increase in secretory output may be due to increased activity per cell and/or increased total L-cell number. It is possible that short-chain fatty acids (SCFA) produced from the fibre fermentation may play a signalling role, since SCFA stimulation of L-cell PYY and GLP-1 secretion has been demonstrated in human and rodent models [23–26]. However, given the greatly increased dimensions of both small and large intestines, it is also highly likely that the total L-cell number along the GI tract was increased, as seen in the typical gut hypertrophy following bariatric surgery [27]. In support, there were significant positive correlations seen here between both PYY and total GLP-1 and weights of small intestine and caecum.

This new rat study corroborates earlier studies in which ingestion of soluble fibre has been reported to alter the physical characteristics of the gut, notably increasing its weight and length [12, 16] and stimulating intestinal mucosal cell proliferation [15]. Other rat studies specifically using dietary pectin also report increased weights of small intestine, caecum and colon, with increased crypt depths and villus heights in jejunum and ileum, and increased intestinal epithelial cell proliferation [14, 28, 29]. Evidence suggests that these physical changes in gut are stimulated by the fermentation products of dietary fibre. In rats, increased soluble fibre, including pectin, increases SCFA production in caecum and colon [2, 30]. Caecal and colonic infusions of SCFAs have been shown to stimulate intestinal mucosal growth and epithelial cell proliferation in rats [31–33], while the proliferative response of the intestinal epithelium to fermentable dietary fibre is abolished in germ-free rats [15].

Although the increases in large intestine size may have been anticipated to accommodate the increased fermentation processes for the pectin-containing diets, additional dose-dependent increases in size were observed for the small intestine. Furthermore, the underlying histological changes (increases in villus height and crypt depth) were most significant in the small intestine, i.e. upstream of fermentation in the caecum/colon, and were therefore unlikely to have been caused by local actions of fermentation products in the lumen. Indeed a similar gut hypertrophy following bariatric surgery occurs without changes in diet composition or hindgut fermentation. Thus Hansen et al observed hypertrophy of the small intestinal gut mucosa in Roux-en-Y gastric by-pass (RYGB)-operated rats [27] and Borg et al reported hypertrophy and increased small intestinal cell proliferation in rats with biliopancreatic diversion (BPD) [34]. In addition, RYGB rats had a doubling of total number of L-cells, with no change in their density, and hence an approximate two-fold increase in intestinal expression of PYY and preproglucagon mRNAs [27], while BPD rats had increased circulating concentrations of PYY, GLP-1 and GLP-2 [34]. While the increases in these hormones are likely a consequence of the L-cell proliferation, which is secondary to gut hypertrophy, they may also be involved in further stimulating the mucosal hypertrophy. GLP-2 is known to potently stimulate intestinal epithelial proliferation [35, 36] and it is synthesised from the L-cell preproglucagon gene in a 1:1 stoichiometric ratio with GLP-1 [37, 38]. Although GLP-2 was not measured directly in the present rats in view of its short half-life and the single terminal blood sampling protocol employed, it is likely that it would have been increased along with the elevated GLP-1 concentrations and could therefore have played an intestinotrophic role in the pectin-fed animals.

Meanwhile, known actions of PYY also include delaying gastro-intestinal transit and slowing gastric emptying [39], and both PYY and GLP-1 are implicated in activating the so-called ileal brake [40]. The resulting longer gut transit times would likely further influence overall rates of digestion and nutrient absorption, with potentially significant alterations in the nutrient milieu to which the intestinal epithelium is exposed along its length. In addition, viscous dietary fibres such as pectin may directly slow transit times via gel formation and may also inhibit absorption of bile acids and minerals [41], thereby further influencing the nutrient profile to which the intestinal epithelium is exposed throughout the gut.

Therefore, significant effects on gut hypertrophy and hyperplasia accompany the desirable effects of soluble fermentable dietary fibre on reduced food intake, body weight gain and adiposity. However, despite the increased weight of the gut, which might be perceived as undesirable, significant reductions in overall body weight gain are still observed. Moreover, gross, microscopic and functional changes in the gut are normal adaptive responses to numerous factors, most commonly diet, but also for example bariatric surgery. In the latter scenario, the intestinal mucosal hypertrophy and crypt cell proliferation are seen as fundamental to the healthy post-operative outcome and sustained weight loss [27, 34, 42].

In conclusion, this is the first report of the dose-dependency of a range of physiological, intestinal and endocrine responses to increased soluble dietary fibre (pectin). It seems from our animal model that increments in daily fibre intake lead to proportional increases in satiety hormones and decreases in food intake and body weight/fat gain, while proportionately increasing intestinal size and mucosal hypertrophy/hyperplasia; these effects are manifest within 8 days and sustained for at least 28 days. It remains to be tested whether similar responses are seen in humans given similar dietary interventions.
